# Use of iPhones by Nurses in an Acute Care Setting to Improve Communication and Decision-Making Processes: Qualitative Analysis of Nurses’ Perspectives on iPhone Use

**DOI:** 10.2196/mhealth.5071

**Published:** 2016-05-31

**Authors:** Maureen Farrell

**Affiliations:** ^1^ University of Melbourne Victoria Australia

**Keywords:** acute care, clinical decision making, communication, iPhones, nursing

## Abstract

**Background:**

Smartphones and other mobile devices are having and will continue to have an impact on health care delivery in acute settings in Australia and overseas. Nurses, unlike physicians, have been slow to adopt these technologies and the reasons for this may relate to the status of both these professions within the hospital setting.

**Objective:**

To explore nurses’ perspectives on iPhone use within an acute care unit. We examined their experiences and views on how this device may improve communication and decision-making processes at the point of care.

**Methods:**

Two focus group discussions, using a semistructured interview, were conducted over the trial period. The discussions focused on the nurses’ experiences regarding ease of use, features, and capabilities of the device. The focus groups were recorded, transcribed, and analyzed using semistructured interview questions as a guide.

**Results:**

The positive findings indicated that the iPhones were accessible and portable at point of care with patients, enhanced communication in the workplace, particularly among the nurses, and that this technology would evolve and be embraced by all nurses in the future. The negatives were the small screen size when undertaking bedside education for the patient and the invasive nature of the device. Another issue was the perception of being viewed as unprofessional when using the device in real time with the patients and their family.

**Conclusions:**

The use of iPhones by nurses in acute care settings has the potential to enhance patient care, especially through more effective communication among nurses, and other health care professionals. To ensure that the benefits of this technology is woven into the everyday practice of the nurse, it is important that leaders in these organizations develop the agenda or policy to ensure that this occurs.

## Introduction

Smartphones and other mobile devices are having and will continue to have an impact on health care delivery in acute settings in Australia and overseas. Nurses, unlike physicians, have been slow to adopt these technologies and the reasons for this may relate to the status of both these professions within the hospital setting. Physicians are often given easier access to the technology needed to run health care apps and administration seems to have a more relaxed attitude toward the use of these devices by these professionals [1,2]. Initially, personal digital assistants (PDAs) [3-16] were first used; however, these have now been surpassed by the smartphone—a PDA with multiple capabilities. These phones offer voice and text communication, advanced computing, and communication capability, including Internet access and geo-positioning systems. Other features include on-board personal management tools, high-quality cameras, and recording devices. Current research suggests that smartphones may support aspects of clinical diagnoses and treatment [17,18], yet there has been limited studies on whether the devices improve decision making and communication at the point of care. The literature that was available highlighted the various means of communication and how these can be used to reduce communication errors [19,20]. Another study suggested that mobile communications may have a negative impact on informal interaction between health care professionals and this may require sociotechnical change [21,22].

Recent systematic reviews highlighted the increased use of smartphones and how they are becoming a necessity for clinical use among health care students and professionals [1,17,23-25]. Real-time evidence-based clinical information was also considered important at the point of care, as it is common for clinicians not to seek answers to clinical queries after completion of a patient encounter [26]. Bedside access to patient information, or access from anywhere, through smartphone use, was accentuated, with a focus on security of personal data [27-29]. Drug reference apps, e-textbooks and references for disease diagnosis and treatment, and medical calculators were reported as most useful for clinicians and students.

The vast amount of medical information, the rapid growth in new pharmacotherapies and technologies, increasing time constraints on clinicians, escalating pressure to reduce costs, and substandard systems for delivery of care make it nearly impossible for clinicians to provide high-quality, error-free care on a consistent basis. In Australian hospitals, the most reported adverse events (ie, incidents in which harm resulted to a patient receiving health care) are infection, falls resulting in injuries, and problems with medication and medical devices [30]. In most cases, a significant number of the errors are the result of faulty system designs and conditions, and not due to individual negligence or incompetence. It is well documented in the literature that mobile technologies have the potential to reduce medical errors or adverse events [20,31,32]; however, there is also the potential for unintended consequences, such as technology-induced errors and distraction [10,33,34]. With the increasing complexity of the health care system, it seems that smartphones, like the stethoscope, are becoming a necessary work tool for clinicians—the challenge is to maximize the benefits of this technology while reducing the negatives.

The registered nurses (RNs) in this study used the smartphone in an acute care unit that treated patients with diseases such as breast and ovarian cancers, obesity, and diabetes, all of which required ongoing support. In Australia, there is limited literature on the use of smartphones in clinical practice, yet it has been embraced in some areas. The expected outcomes of this study are improved communication and decision-making processes within the acute care unit by nurses and other health care professionals. This may indirectly lead to more efficient health care delivery, with reduction in costs related to medical errors, especially in the area of medication prescribing and administration. Increased efficiency in execution of patient care may result as the nurses will have more time to provide direct patient care. The smartphones can also be used for education of patients, especially those with diseases that require ongoing support. There may also be a cultural change within the unit, that is, nurses and other health professionals embrace the use of these mobile technologies, so that they are embedded in the workplace and in the workflow.

## Methods

### Study Objective

This study used both quantitative and qualitative methods to investigate the use of smartphones by nurses in an acute care setting. This paper focuses on the qualitative data that were captured in focus group discussions to explore the nurses’ perspectives on the use of smartphones for communication and decision-making processes.

### Sample and Setting

The research was undertaken in the Gynecological Ward at Royal Women’s Hospital (Melbourne, Australia). A total of 20 RNs were purposively selected to participate in the project. All received an 8-GB iPhone 5 (mobile device selected for this study), a waterproof case, and an iTunes card to the value of AUD $30 (donated by Apple Australia) to download relevant software apps—MIMS Drug Information, MIMS Drug Interact, MedCalc, and PubSearch. The key features of the device included a 4-in. Retina display, an Apple-designed A6 chip for faster performance, and a longer battery life. JB Hi FI and Telstra, the vendors for this project, provided the equipment and a AUD $60 monthly plan for each nurse. They also conducted a 2-hour training session for the participants in iPhone use at the commencement of the study and provided ongoing support for any technical issues, if needed, throughout the 12-month trial period. To ensure that the AUD $60 plan was not exceeded by the nurses, Telstra provided tracking of the iPhone by text messages and voice usage. The Human Research Ethics Committee of the Royal Women’s Hospital approved the study.

### Data Collection and Analysis

A semistructured interview was used in the focus group discussions. The participants were asked to describe their general impressions of the iPhones; any impediments or barriers encountered when using the iPhones; support they received from the training team; impact of the iPhones on their communication processes with other nurses and health care professionals, on their decision making, and on the culture within their work environment. The audiotapes were transcribed verbatim and the content was analyzed using the semistructured interview as a guide for identified themes.

## Results

The narrative data were analyzed around the questions used in the semistructured interview with scope for follow-up questions.

### General Impression, Impediments, and Barriers Encountered When Using the Mobile Device

Most of the nurses found the iPhones easy to use as reflected in the following statement:

A lot of people who’d already used one [an iPhone] picked it up quite quickly.

The physical size of the screen was problematic and was deemed too small to use at the bedside and to research information. The speed processing and graphics were reported as fine yet battery life was reported as problematic for some nurses. The main reason for the latter was problems with the charger for some of the devices. They were faulty and had to be replaced by the vendors. Others were simply not recharging the device at home, and thus, to overcome this limitation, some nurses brought a charger to work. With regard to the data usage, there were inconsistencies in how often this was checked. Some respondents checked their usage regularly, whereas others did not know how to do so. Consequentially, a participant reported going over the data allowance and identified the problem to be related to connection error with the iPhone,

I went over on my data allowance...because it wasn’t picking up the Wi-Fi network here.

The main impediments reported for using the iPhones were reported as the physical size of the screen, connectivity issues, commonality for American apps to be more available/accessible, and the potential generation gap in technology. Various nurses announced these specific accounts for these evidential barriers. For example, as one nurse noted,

The actual size of the phone and screen...too small. I think a printout is easier.

There were repeated responses regarding the preference to hardcopies for patient education, *I don’t think there’s a demand for it in your patient care...and that you need to be using the Internet to educate the patient. We’ve got booklets from the Cancer Council and things like that...I’d much prefer to give to a patient*

If they’ve got a physical booklet in front of them, it’s going to be a lot better than a website.

With regard to connectivity issues, one nurse said,

I couldn’t load up the bundle...the website I need...I think there’s a security control here.

Another challenge certain nurses experienced was the difficulties in finding Australian-normed apps instead of American (with reference to drug calculations),

A lot of it is American...there’s acronyms for things that I haven’t heard of...I’m struggling to find an Australian standardised app.

The theme of the generational gap emerged from this nurse,

I think specifically the older nurses, who haven’t used or who haven’t had an iPhone were thrown in the deep end...and went this is too hard.

The final and potentially most pronounced theme was the nurses being perceived as unprofessional, which inhibited and discouraged the iPhone use, especially at the bedside, as reflected in these statements:

Doing your own personal stuff on work time when you should be looking after their relative or their family member.

I’ve found I have to explain that it’s a work phone...because I feel it looks unprofessional.

I feel like it’s rude to them [patients] to be on the phone.

Similarly, a nurse noted that the physical factor of having a phone with you could consequentially have negative implications as “it can intrude on those conversations.”

### General Impressions About the Support From the Training Team

The majority of the respondents stated that they did not receive enough training and support at the initial stages of the study. Downloading the software apps was problematic for some of the nurses and believed that this should have been done before they had received the device. In addition, some nurses reported technical complications relating to login details

It took a whole month for me to figure out all the logins.

Some also noted a *lot of teething issues* at the commencement of the project. In terms of ongoing support, certain nurses disclosed that it was a matter of practice and *“getting used to the iPhone,”* whereas another nurse noted they require “continuous education.” The nurses supported each other as noted in the following response:

I contacted IT and they helped me and so I was able to sort of set everyone else up.

Similarly, there appeared to be a collaborated effort with regard to the types of apps downloaded,

Someone downloads and says that’s a good app, so all the others download it as well.

The good apps cited by the nurses were BreCan (patient education, nursing needs, and updated clinical information), Coloplast (wound care and dressing selection), LactMed (medications that can be used when breast feeding), MIMS (pharmacological database for checking drugs), and MedScape (point-of-care decision making including drug administration). With regard to iTunes accounts there were inconsistent reports of set ups as exemplified by the following statements:

Everyone set up the iTunes account, so there are a few things downloaded.

Half of them didn’t have an iTunes account.

Only 1 respondent stated seeking assistance from Apple, where they went into an Apple store and downloaded apps with assistance in the store.

### Impact of iPhones on Communication Processes With Other Nurses and Health Care Professionals

Communication was the dominant reason for iPhone use. One respondent noted,

I would think 95% of the time it’s for communication between staff.

The most prevalent type of communication was phone calls and texting, depicted with the following responses,

I would say the majority of the time I use them for calling.

*I would text more often...but not always...because it looks like you’re texting socially*.*”* One participant reported the issue of response time with text messages,

They don’t get answered as fast...don’t know if they got it.

Participants also reported using the iPhones to check their emails: *getting emails has also been useful.* There were varied responses with regard to how often the nurses used the iPhones; however, the average was around 2-4 times/shift. The general consensus among the respondents was that communication between nurses had improved,

It’s very good communication between us.

I think it has been really good communicating between staff.

The nurses reported that the main reasons for making contact with other nurses was centered on changing shifts, social agendas, and checks related to patients. There were no reports of using the iPhones for organizing professional development sessions, although some of the nurses reported that they had organized meetings for staff via the iPhone email or arranged meeting points using the device.

Communication with other health care professionals yielded varied responses:

I haven’t used it to page any doctors or anything though.

Sometimes I think it’s easier to page them by their phone.

By contrast, effective use of the device was also reported, as one nurse noted,

I’ve called people directly...leave my number and the doctor’s call me back.

The most common reasons for contacting doctors included medication and intravenous (IV) fluid orders and reviews. In terms of these health care professionals’ responses, most nurses appeared to incur positive feedback, as the following inferences indicate,

Some doctors are on board...[doctor] called me on my iPhone.

Their awareness of it is good as well, for them to be able to contact us.

Ward clerks and the nurse in charge use it to contact you if your patient needs to go to imaging or needs to be transported.

Yet other nurses had experienced less positive encounters and felt that there was a lack of knowledge about the project, as one key informant implied,

A lot of clerks still yell for me.

Similarly, the nurses reported inconsistent experiences with speed of reply from doctors,

I needed a fluid and blood order for a patient and I paged one of the doctors. It had been a good half an hour so I paged again and nothing. So I found another doctor and then they got back to me straightaway.

There was a continuous theme that emerged throughout the interviews that indicated the project was a transitional process, to which the nurses and other staff adapted,

At the start of the trial the doctors were quite unsure of it...because they weren’t told about it...seem fine with it now.

There was frequent reporting that other health care professionals (most commonly referring to doctors) use similar technologies:

They use their phones all the time.

Look up their phones a lot more than us.

The doctors download this app and you communicate between the apps, and that’s the paging system.

This example highlighted the notion that doctors are already utilizing technological advancements efficiently.

In the case of a clinical emergency, the first reaction from the respondents was generally not to use the iPhone,

I don’t think it [iPhone] would be realistic to ever use in an emergency, because with bedside phones it’s a four digit number...so it’s just easier to use the bedside phones.

However, with further deliberation, the nurses disclosed the benefits of the iPhone if they did not have access to a phone, as reflected in the following statements:

We had a patient with something going on with their heart...Paged [doctor] and they called back within 30 seconds...on the ward within two minutes. That was really good...and we didn’t have to leave the patient.

If you were somewhere else in the hospital, not near a phone...probably handy... in a situation where, just say you were down the car park and its dangerous or in the lift, and you’re by yourself and something happens.

### Impact of iPhones on Decision Making and Culture Within the Work Environment

Very few nurses reported using decision-making software apps when providing care. The common reason was generally based on the habit of going to a computer, as indicated in the following comments of key informants,

I still find myself going to the computer to look at that [MIMS], it’s just probably more habit.

I just find it quicker to jump on the computer and I did MIMS today. I totally forgot about the iPhone.

The nurses reported that they had experienced a variety of responses from patients regarding the nurses using their iPhones for direct care. There was a continuous theme of the nurses experiencing a degree of unease when using the iPhones at the bedside, and noted the inappropriateness of answering calls during particular times as the following nurse articulated,

I always think when I ring another staff member and they’re in with the patient...having discussions about dying and death...then the phone goes off...if I was talking and giving some education [to a patient] it would be so rude...to answer my phone.

The majority of nurses stated that the small screen size of the phone made it inappropriate to use at the bedside, especially with older patients:

If someone is elderly...showing a tiny little screen about the website or something...I think a lot of our education and information for patients is a print off that they can take home and read. It’s not something you sit there and go through or flick through on the phone with them.

Yet some did admit that they *“looked a few things up”* when they needed information quickly or they did not have access to a computer.

Furthermore, with regard to cultural change, several nurses reported that the iPhones were the beginning *“of what’s to come”* and were integral to modernizing the workplace. For example, one nurse remarked,

The culture today is that everyone is on the phone...it does save a lot of time. It has the potential to be brilliant. I think the biggest concern for all of us was that we didn’t get a really good set up at the start.

There was also the belief that there was a degree of evolution and gradual change in culture as exemplified by the following comments from nurses:

Things just take time to sink in, I think this will be more useful gradually. Once we use it more and get used to use it. I think it’s the future, or it’s the current future. Have to get into this.

Junior nurse might use the Internet a lot on their iPhone as compared to the senior nurse who has a lot more knowledge. So it might be really helpful for a junior nurse.

Another respondent stated that

She had been nursing for nearly nine or 10 years [and] never had this opportunity really to have a phone and to look up apps and to show patients that

One nurse with reference to technological advancements believed that a device with a larger screen, such as an iPad, would be next to every bed in the future. Others shared this sentiment,

Now they’re talking about iPads...bigger screen...that’d be good for education.... I’d feel more comfortable showing them something on an iPad more than getting out my tiny little phone.

However, some reported that

iPads are not good for communication because you can’t call on it or put it in your pocket.

## Discussion

### Preliminary Findings

Communication and decision-making issues among health care professionals are a major problem in the clinical area and often result in medical errors, that in most cases, are preventable [19,20]. The use of iPhone by nurses to address this problem identified varying themes that are related to practical use, impediment and difficulties encountered, impact the devices had on their communication and decision-making processes, and future use. As most of the nurses were from the Generation Y age group, they found the iPhones easy to use and had no problem with speed processing and graphics, yet staying within their daily data allowance. Problems encountered were the small size of the screen and the battery life, findings that were consistent with similar previous studies [35,36]. It is interesting to note that screen size was a problem; however, this was related to downloading PDFs or information that was difficult to read, not to apps that had been specifically designed for iPhone. Another impediment articulated by most of the nurses was being perceived as unprofessional when using the device with the patient or family at the bedside. They felt it was rude to answer the iPhone or attend to a text when administering direct care or speaking to family members. This has been identified in other studies where nursing and allied health professionals found it disruptive when doctors answered calls during interprofessional rounds [21,37]. However, explaining the reason for using a mobile device to the patient was found to have a positive effect on patient-physician interactions and communications [38]. In this study, the nurses did inform the patients and their family that it was a work iPhone and there was a flyer in each room stating that the nurses would be using the iPhone to assist them with the decision-making and communication processes. With regard to support from the training team, most nurses reported that this was inadequate at the beginning of the trial period, and so they sought assistance from each other to find solutions to the problems encountered (eg, downloading software apps). Ongoing support was not required as most nurses improved with practice and became familiar with the use of the iPhone.

Communication was identified as the major reason for iPhone use with the most common medium being phone calls and texting, undertaken at least two to four times/shift. The general consensus was that communication between nurses had improved with the focus being on checks related to patients, changing shifts, and social agendas. Contacting doctors and other professionals within the work environment varied, as some used the iPhone whereas others still used the paging system. The most common reasons for contacting doctors were for IV fluid orders and medication reviews. Clinical emergencies at point of care were not a high priority for iPhone use as there was a bedside phone; yet, most nurses acknowledge that they would use the device if they did not have access to this phone, and this was the case for some patients with cardiac problems. Others stated that they would use the iPhone for their own personal safety, for example, in car parks or trapped in lifts. The ward clerks and the nurse in charge also contacted the nurses on the iPhone mainly for transportation of patients to imaging or other areas within the hospital, and most believed this was very beneficial. What did emerge from these narratives, which is significant, was the prolific use of smartphones by physicians. All the nurses recognized how far advanced they were in the use of these technologies, not only for efficiencies in clinical practice, but also for communication among themselves. This finding is consistent with the literature reports [1,2].

Using the iPhones for decision making and educating patients/family at the point of care was not fully embraced by all nurses. Most felt a degree of unease, as stated previously, and were inclined to look up information on the ward computers rather than the iPhone. When educating patients/family, the main issue was screen size, especially for older people, and the print material was more accessible. Yet at the end of the trial period most agreed that they did use the iPhone to look up information quickly, particularly when the ward computers were being used by other health care professionals. The information most sought was related to drug administration, which is evidenced by the literature [2]. When asked about future use of iPhones, most agreed that it will only be a matter of time before these technologies are integrated into their practice, as a necessary clinical tool. Some believed the iPad would be more beneficial as it had a larger screen; however, others reported that it was not as accessible and mobile as the smartphone.

These findings suggest that iPhone will be adopted by nurses in clinical practice, primarily for communication between themselves and doctors. Although the device was not used as much for decision making, this will evolve and the ideal software apps developed will need to assist with workflow, offer quick information about medications, illnesses, or symptoms, and coordinate multiple activities.

### Conclusion

To our knowledge, this study is significant, as it is the first in Australia to comprehensively investigate the use of iPhones by nurses in acute care unit to enhance communication and decision-making processes. As stated previously, this study focused on the qualitative aspect where the nurses’ perspectives on iPhone use within this environment were explored. The most significant themes that emerged from the narratives were that all the nurses embraced the use of iPhones and believed that the device will become a necessary clinical tool, like the stethoscope. This is a promising finding as nurses, unlike physicians, have been slow to adopt mobile technologies in Australia and overseas. The challenges now lie with nursing leaders and managers, in both the education and clinical sectors, to ensure that these technologies are adopted. Overall, it is clear that more research and development are needed to fully realize the potential benefits of these technologies, especially the impact on patient health outcomes.

## Abbreviations

IV: intravenous
PDAs: personal digital assistants
RNs: registered nurses
